# Supplementary material for: A Global Screen for Assembly State Changes of the Mitotic Proteome by SEC-SWATH-MS
Source: Cell Syst. 2020 Feb 26;10(2):133–155.e6. doi: 10.1016/j.cels.2020.01.001 (PMC7042714; doi:10.1016/j.cels.2020.01.001)

Q8IWR0 | Z3H7A_HUMAN | ZC3H7A ZC3H7 ZC3HDC7 HSPC055
Monomer MW [kDa]: 110.538 Monomer expected elution fraction: 39

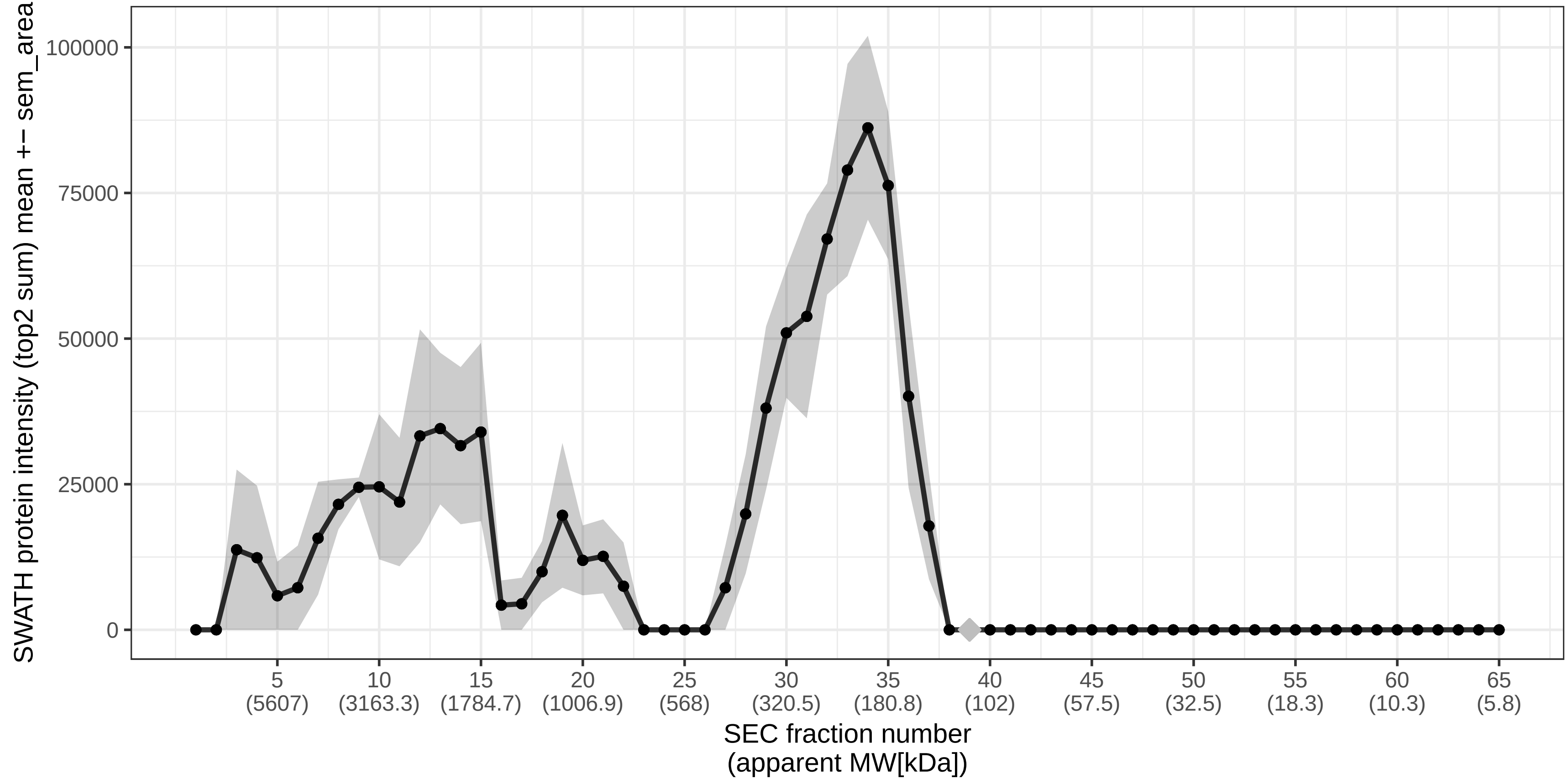

Supplement: Data S1. SEC-SWATH-MS Protein Chromatograms, Related to Figure 1 [file mmc6.zip › SECchrom_Q8IWR0_Z3H7A_HUMAN_ZC3H7A_ZC3H7_ZC3HDC7_HSPC055.pdf]
